# Rosai-Dorfman disease mimicking lung cancer: a case report

**DOI:** 10.3389/fonc.2025.1663537

**Published:** 2025-10-15

**Authors:** Caijuan Zhang, Ruoling Gao, Zhenwei Chen, Zhilan Huang, Tian Yang, Ji Bo Hu

**Affiliations:** ^1^ Department of Radiology, The Fourth Affiliated Hospital of School of Medicine, and International School of Medicine, International Institutes of Medicine, Zhejiang University, Yiwu, China; ^2^ Department of Pathology, The Fourth Affiliated Hospital of School of Medicine, and International School of Medicine, International Institutes of Medicine, Zhejiang University, Yiwu, China

**Keywords:** Rosai-Dorfman disease, lung, IgG4-related disease, histiocytosis, radiological presentation

## Abstract

**Background:**

Rosai-Dorfman disease (RDD) is an uncommon, benign histiocytic condition with an unclear underlying cause. It typically manifests as painless, bilateral, and significantly enlarged cervical lymph nodes. However, extranodal involvement can also occur, affecting the skin, nasal cavity, bones, orbital tissues, and central nervous system (CNS). Moreover, involvement of the respiratory system is observed in approximately 2% of cases.

**Case description:**

A 75-year-old female was admitted to our hospital with a one-week history of lumbar and back pain. Chest computed tomography (CT) identified a nodule located in the lung upper right lobe, characterized by spiculated margins and proximal bronchial atresia, raising suspicion for lung cancer. The patient subsequently underwent thoracoscopic lobectomy. Postoperative histopathological examination and immunohistochemical analysis confirmed the diagnosis of RDD.

**Conclusion:**

Pulmonary RDD is rare and can be easily mistaken for other tumors or inflammatory diseases because of its nonspecific clinical and radiological features. Definitive diagnosis requires histopathological evaluation and immunohistochemical analysis.

## 1 Introduction

Rosai-Dorfman disease (RDD) is an infrequent histiocytic condition with an undetermined cause. Initially documented by Paul Destombes in 1965, it was later thoroughly characterized by Rosai and Dorfman in 1969 under the term “sinus histiocytosis with massive lymphadenopathy.” (1, 2) According to the 2016 expert consensus of the Histiocyte Society, RDD is classified within the “R group” of histiocytoses (3) and has traditionally been considered a benign, self-limiting disease. However, recent evidence has demonstrated that a subset of cases harbor gene mutations involving the MAPK/ERK pathway, suggesting a neoplastic rather than purely reactive process (4). Reflecting these insights, RDD was designated a histiocytic neoplasm in the 5th edition of the WHO Classification of Haematolymphoid Tumors, published in 2022 (5).

Although RDD can develop at any age, it predominantly affects children and young individuals. It can be classified into three types: purely nodal, purely extranodal, and combined nodal and extranodal disease (6). Classic cases typically exhibit extensive, symmetrical, and non-tender enlargement of the cervical lymph nodes. Extranodal sites are frequently affected, including the skin, nasal cavity, bones, orbital tissues, and CNS (4), while involvement of the respiratory system is rare, occurring in only approximately 2% of cases (7). Here, we report a case of a pulmonary manifestation of RDD, presenting as a solitary pulmonary nodule, and discuss the clinicopathological and radiological features of pulmonary RDD.

## 2 Case report

A 75-year-old female initially sought medical attention at an external facility with a one-week history of lumbar and back pain. Thoracolumbar magnetic resonance imaging (MRI) revealed compression fractures of the 11th thoracic vertebra and fractures of the spinous process of the 10th thoracic vertebra. Chest CT imaging revealed a soft tissue nodule located in the lung right upper lobe (RUL). The patient was later referred to our hospital for comprehensive assessment and treatment planning. She did not report any chest tightness, chest pain, fever, or weight loss. Her medical history was notable for chronic gastritis and prior uterine fibroid surgery. Laboratory tests showed mildly elevated levels of cytokeratin 21-1 (2.77 ng/mL) and carbohydrate antigen 72-4 (19.1 µg/L).

**Figure 1:**
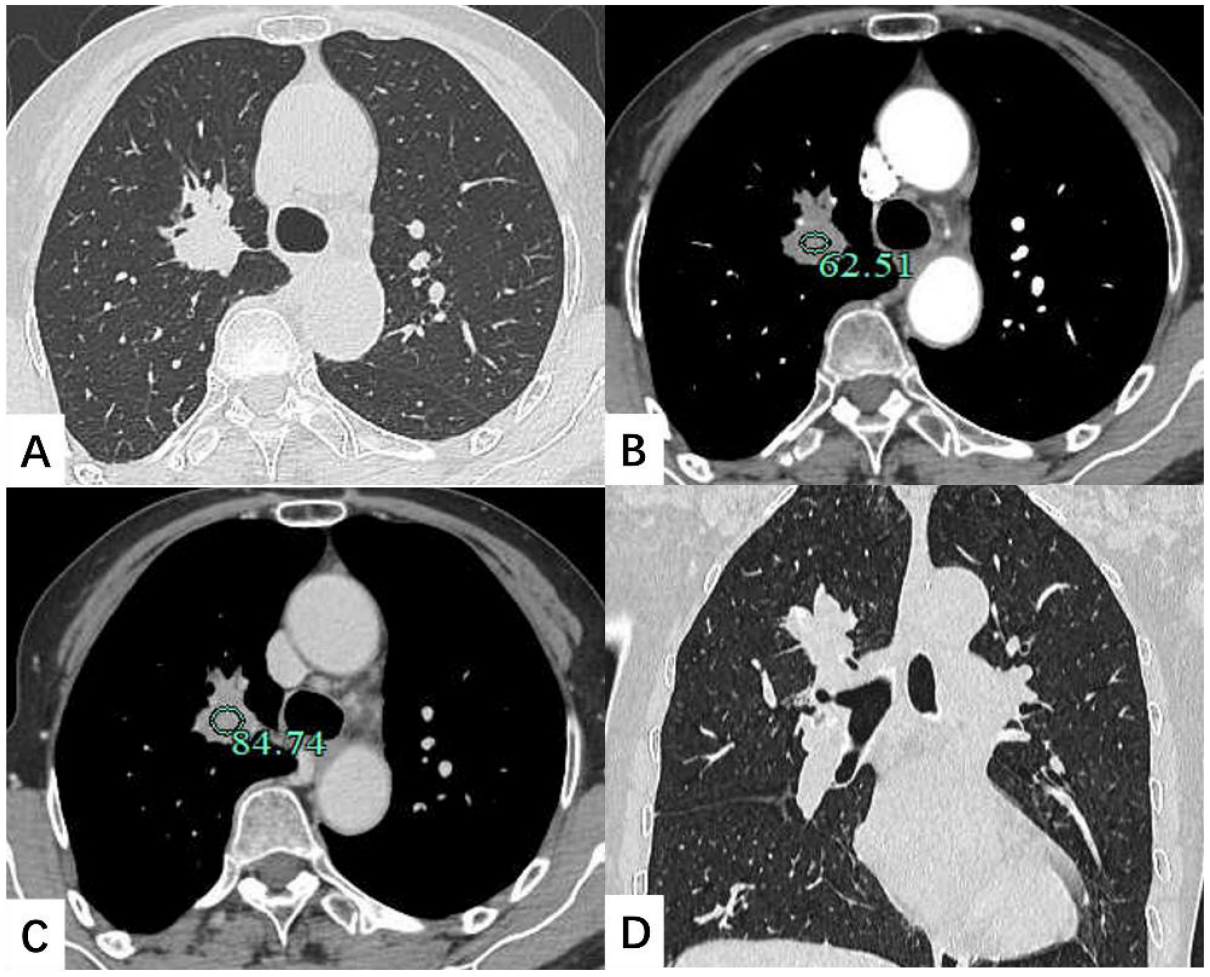
Contrast-enhanced chest CT scan depicted a 30 × 25 mm soft tissue nodule located in the lung right upper lobe. The shadow exhibited mild enhancement on the enhanced scan. **(A)** Transverse position CT plain scan; **(B)** Transection CT scan arterial phase; **(C)** Transection CT scan venous phase; **(D)** Coronal position CT scan.

A contrast-enhanced chest CT scan revealed a 30 × 25 mm nodule in the lung RUL, characterized by spiculated margins and proximal bronchial atresia, findings suggestive of lung cancer (**Figure 1**). Enlarged right hilar lymph nodes were also observed. Bronchoscopy demonstrated obliteration of the tip segment of the lung RUL, with mucosa appearing edematous. Endobronchial ultrasound revealed hypoechoic shadows within the right lung that exhibited uniform internal echoes. Bronchoscopic biopsy showed fibrous exudate containing scattered lymphocytes and glandular epithelial cells.

**Figure 2:**
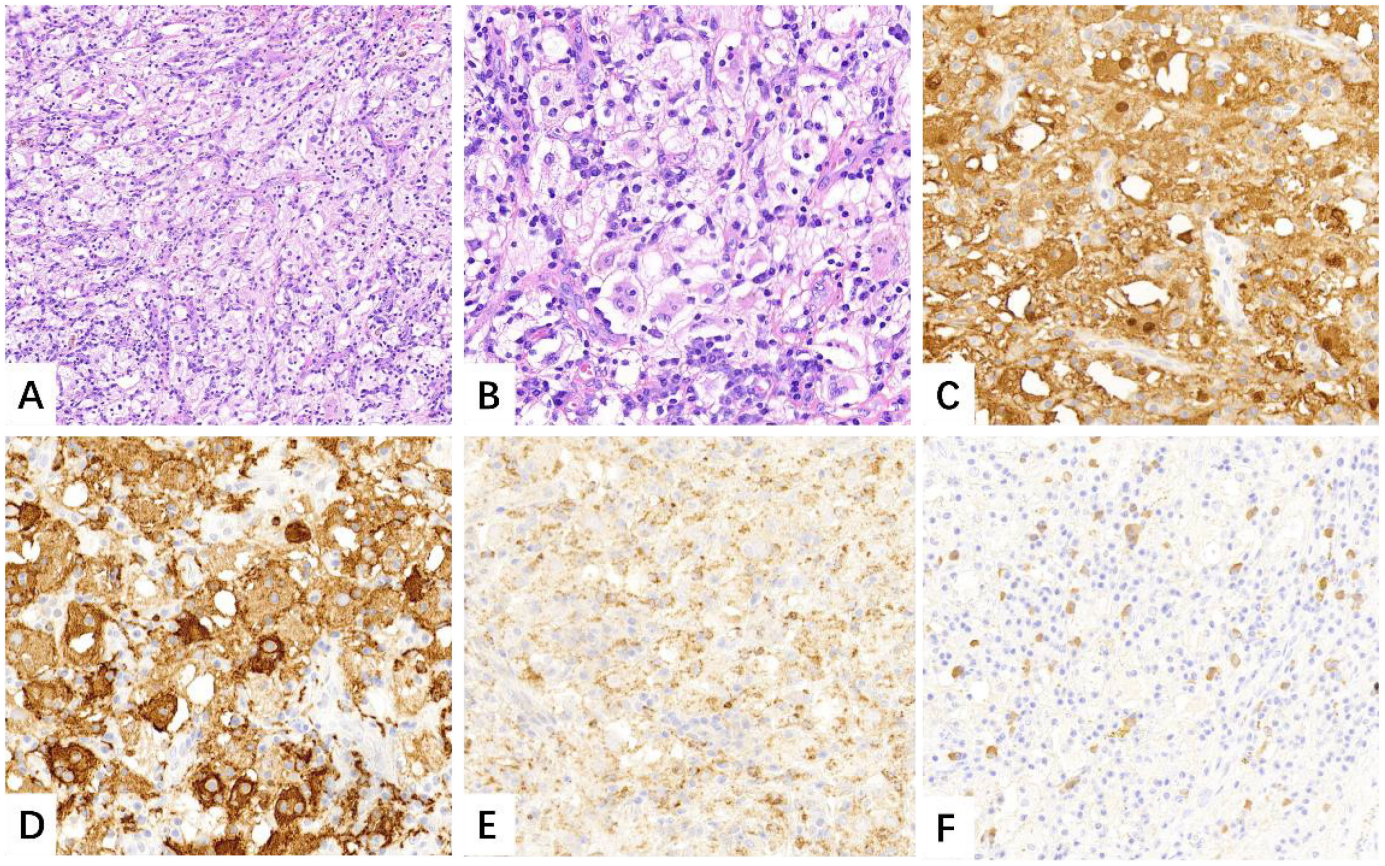
Numerous large histiocytoid cells were seen within the lesion that admixed with lymphocytes and plasma cells [**(A)**, H&E, ×10]. High magnification revealed evidence of emperipolesis [**(B)**, H&E, ×20]. Immunohistochemistry showed S-100, CD163 and CD68 positively [**(C–E)**, IHC, ×20]. Focally increased IgG4-positive plasma cells were also noted [**(F)**, IHC, ×20].

Three days after admission, the patient underwent thoracoscopic lobectomy. Histological analysis revealed fibrous tissue proliferation with infiltration of numerous lymphocytes, plasma cells, and histiocytes within the interstitium. On higher magnification, some histiocytes demonstrated emperipolesis, engulfing lymphocytes and plasma cells. Immunohistochemically, the histiocytoid cells were positive for S100, OCT2, CD68, CD163, and Cyclin D1. Immunohistochemical staining for ALK, STAT6, and cytokeratin (AE1/AE3) was negative. The lung specimen also demonstrated aggregates of increased IgG4-positive plasma cells (**Figure 2**). All 15 lymph nodes submitted for evaluation showed reactive hyperplasia. The histopathological and immunohistochemical findings were consistent with RDD. During the subsequent five-month follow-up period, the patient exhibited no symptoms. Moreover, there were no signs of tumor recurrence, metastatic spread, or involvement in other organs.

## 3 Discussion

RDD is a rare form of histiocytosis. The predominant clinical presentation is painless cervical lymphadenopathy, bilateral, and massive. However, approximately 43% of cases present with extranodal disease, with or without concurrent nodal involvement (8, 9). Intrathoracic involvement is rarely reported in patients with RDD and can be classified according to the primary site of involvement as pulmonary, mediastinal, pleural, or airway disease (10). Mediastinal lymphadenopathy is the most common intrathoracic manifestation, followed by pulmonary involvement. The clinical features of pulmonary RDD are nonspecific and may range from asymptomatic presentations to progressive dyspnea, or, in rare cases, acute respiratory failure.

The radiological presentation of pulmonary RDD is heterogeneous and can easily be mistaken for other neoplastic or inflammatory diseases. Imaging findings may include solitary or multiple nodules, interstitial lung disease, ground-glass opacities, and peribronchovascular consolidation, with or without associated lymphadenopathy (7, 10–12). These CT features of lung involvement may coexist in the same patient. The presence of cysts has been rarely reported in pulmonary RDD. Moyon et al. (10) suggested that cyst formation might be associated with Langerhans cell histiocytosis, whereas Goupil et al. (7) proposed that it resulted from histiocytic infiltration of lymphatics along the broncho-vascular bundles and subsequent bronchiolar obstruction. In this case, imaging revealed a solitary pulmonary nodule with spiculated margins and proximal bronchial atresia, which was difficult to distinguish from lung cancer.

Pulmonary RDD has no specific clinical symptoms or characteristic imaging findings, hence a definitive diagnosis requires confirmation through histopathological evaluation. Histologically, RDD is characterized by abnormal proliferation of large histiocytoid cells with abundant eosinophilic cytoplasm, prominent nucleoli, and vesicular nuclei. The existence of lymphophagocytosis within the cytoplasm of histiocytes, known as emperipolesis, is characteristic of RDD, although it is not required for diagnosis (9). Immunohistochemically, the histiocytoid cells typically show positivity for CD68, CD163, S100, cyclin D1, and OCT2 (13, 14). In our case, all diagnostic criteria for RDD were fulfilled.

With the increasing understanding of RDD and IgG4-related disease (IgG4-RD), it has been observed that a subset of RDD cases are accompanied by elevated numbers of IgG4-positive plasma cells or demonstrate overlapping features with IgG4-RD (11, 15, 16). Reports have described similarities in clinical manifestations, anatomical sites of involvement, histopathological features, and treatment approaches between these two diseases (17). Consequently, some authors have speculated that RDD and IgG4-RD may represent conditions within the same disease spectrum, or that IgG4-RD features may reflect a particular phase of RDD (18, 19).

However, most scholars remain cautious regarding the relationship between RDD and IgG4-RD. Wang et al. (20) investigated the pathological characteristics of seven patients with RDD mimicking IgG4-RD and found that none exhibited obliterative phlebitis or storiform fibrosis, suggesting that these specific pathological features are reliable criteria for differentiating between the two diseases. Conversely, other reports have described cases of RDD demonstrating obliterative phlebitis and storiform fibrosis (21). which further complicates the differential diagnosis. In our case, there was marked fibrous tissue hyperplasia and an increased number of IgG4-positive plasma cells. However, the results were insufficient to fulfill the diagnostic criteria for IgG4-RD, and the postoperative serum IgG4 level was within the normal range. The definitive method for distinguishing RDD from IgG4-RD, as well as their precise relationship, remains controversial. Further prospective studies with larger sample sizes are essential to clarify these issues.

RDD can involve multiple systems. In this case, the patient initially presented with lumbar and back pain, and MRI at an external institution revealed vertebral compression fractures. However, the patient and her family declined surgical treatment. A limitation of this case was the lack of imaging data and pathological confirmation. Bone involvement in RDD typically appears as lytic lesions on radiographs, often aggressive in nature, with cortical destruction and periosteal reaction. Vertebral involvement may present as vertebral collapse or flattening on imaging (22). Therefore, vertebral compression fractures caused by RDD cannot be excluded in this case. Ongoing follow-up is planned.

There is currently no standardized treatment for RDD; management is determined by the degree of disease involvement and the patient’s clinical condition. Surgical resection is the preferred approach for isolated pulmonary RDD. Systemic therapy may be indicated for patients with multifocal extranodal involvement that cannot be surgically removed, including corticosteroids, sirolimus, radiotherapy, chemotherapy, or immunomodulatory agents (9). In recent years, postoperative genomic analysis and targeted therapies have offered new treatment directions for RDD.

Although RDD typically follows a benign course, about 10% of patients may die of their disease due to direct complications, infections, or amyloidosis (23, 24). Some reports have suggested that pulmonary RDD may be associated with a worse prognosis (25). However, the prognosis of pulmonary RDD is not well defined because of the small number of reported cases, variable treatment approaches, and limited long-term follow-up data. In our case, given the patient’s stable condition, annual chest CT scans were recommended to monitor disease progression.

## 4 Conclusion

We have reported a rare case of pulmonary RDD presenting as a solitary pulmonary nodule on CT imaging, mimicking primary lung cancer. The clinical and radiological manifestations of RDD are heterogeneous, which makes the disease susceptible to misdiagnosis or missed diagnosis. Definitive diagnosis typically relies on histopathological evaluation. Greater awareness of the diverse imaging features of pulmonary RDD may facilitate more accurate clinical assessment and improve differential diagnosis.

## Data Availability

The original contributions presented in the study are included in the article/Supplementary Material. Further inquiries can be directed to the corresponding author.
